# Supplementary material for: Cost analysis of the TB-PRACTECAL clinical trial on novel tuberculosis treatment regimens
Source: PLOS Glob Public Health. 2025 Apr 23;5(4):e0003759. doi: 10.1371/journal.pgph.0003759 (PMC12017904; doi:10.1371/journal.pgph.0003759)
Supplement: S1 Text — (DOCX) [file pgph.0003759.s001.docx]

**S1 Text: Appendix**

Background. MSF clinical trial transparency policy (extract relevant to clinical trial costs)

Table A. Overview of TB-PRACTECAL trial sites.

Table B. Cost categories, definitions, and notes on rationale.

Methods. Systematic review of available publications on the cost of clinical trials.

Table C. Database search strategy

Results. Literature identified in the systematic review.

Figure A. PRISMA flow diagram for literature search.

Table D. Table of characteristics of publications included in the systematic review

Table E. Per-patient costs.

Table F. Costs by category, per patient (EUR).

Table G. Costs by category, per patient, as percent above/below the average.

Table H. Top 10 trial medicines by total cost.

References

**Background. MSF clinical trial transparency policy (extract relevant to clinical trial costs).**^1^

Publication of clinical trial costs

Clinical trial costs are the largest contributor to total research and development costs and poor reporting practices hinder the creation of reliable cost estimates and proportionate funds for research and innovation.^2^ By reporting disaggregated cost data for clinical trials, MSF allows other entities to generate reliable predictions for future R&D ventures and make proportionate and rational investments – especially for research in low-resource settings.

Because costs associated with conducting clinical trials differ on a case-by-case basis and are often difficult to disaggregate from other ongoing operational costs, this policy cannot define exact reporting requirements. However, drawing on published reporting guidance, the following cost items should be disclosed publicly, wherever pragmatic and possible.^3^

Cost items to be reported for the overall study in all cases. All costs imputed to a clinical trial as per budget validated:

- Personnel costs (including salary and benefits for all staff dedicated to clinical trial implementation)
- Costs of external validation of samples/external procedures (clinical procedures including clinical examinations)
- Costs of drugs/laboratory items or any other related material specific to the clinical trial, transport and travel costs if relevant
- Publication Costs, Subawards/Consortium/Contractual Costs
- Cost items to be calculated as an approximation:
- HQ costs: time allocated to follow up by experts including legal advice, data protection, MSF ERB and other services when relevant
- HR costs of field staff supporting clinical trial implementation as part of their project management functions
- If the clinical trial is embedded in an ongoing project “a cost estimation for those patients in the project involved in the Trial”
- Resources allocated for specific community engagement activities

**Table A. Overview of TB-PRACTECAL trial sites.**

| **Trial site** | **Enrolment** | **Dates of activity*** | **Partner organisations and subcontractors** |
| --- | --- | --- | --- |
| Belarus |  |  |  |
| Minsk | 99 | Oct 2016-July 2023 | Ministry of Public Health, Republic of Belarus |
| Uzbekistan |  |  |  |
| Tashkent | 55 | Dec 2015-May 2023 | Ministry of Health, Republic of Uzbekistan |
| Karakalpakstan | 193 | March 2015-Jan 2023 | Ministry of Health, Republic of Uzbekistan |
| South Africa |  |  |  |
| THINK | 163 | May 2017- Dec 2022 | THINK TB & HIV Investigative Network |
| Wits (Durban) | 30 | Jan 2019-Feb 2023 | Wits Health Consortium (Pty) Ltd |
| Wits (Johannesburg) | 12 | Jan 2019-Feb 2023 | Wits Health Consortium (Pty) Ltd |
| Total | 552 |  |  |

*Trial activity is defined here as any investment in work at the trial site, not enrolment.

**Table B. Cost categories, definitions, and notes on rationale.**

| **Cost categories** | **Definition** |
| --- | --- |
| **1. Central activities** | Central activities – also sometimes referred to as sponsor activities – are expenditures incurred by the trial sponsors and include support to sites, as well as expenditures on services that support all sites, including trial planning, day-to-day trial management, administration, statistical analysis, adverse effect monitoring, laboratory quality assurance and control. |
| 1.1 Trial planning (protocol development etc.) | All central costs which are incurred prior to the randomization of the first patient and are not incurred by the sites themselves. These costs do not include transport/travel, purchase of materials or community engagement. |
| 1.2 Regulatory compliance | Trial costs which are related to trial and/or pharmaceutical regulatory requirements. |
| 1.3 Trial management | Trial management costs, including staff time required for day-to-day management and administration. |
| 1.4 Data management | Costs related to storage, cleaning, dissemination and other management of subject and trial data as well as staff time allocated to this end. |
| 1.5 Trial monitoring (including lab and clinical SDV) | Costs related to laboratory and clinical source data validation as well as staff time allocated to this end. |
| 1.6 Pharmacovigilance (safety reporting) | Costs related to safety reporting related to the investigative medical product in question as well as staff time allocated to this end. |
| 1.7 Analysis of results, publication | Staff time allocated to the analysis of results data, manuscript preparation, publication submission processes, presentation of results at conferences etc. |
| 1.8 Central activities - not allocable to below categories | Not allocable office costs, not related to site costs. |
| **2. Trial site staff costs** | Staff costs incurred by sites – sometimes also referred to investigator costs. |
| 2.1 Trial site staff (specifically contracted) | Personnel costs (including salary and benefits for all staff dedicated to clinical trial implementation on trial sites) |
| 2.2 HR costs of field staff supporting clinical trial implementation as part of their project management functions | HR costs of field staff supporting clinical trial implementation as part of their project management functions |
| **3. External services supporting work at clinical trial sites** | Costs related to outsourced/contracted activities. This may differ greatly depending on the trial and trial partners. |
| 3.1 External clinical procedures | Cost of external procedures (clinical procedures including clinical examinations) |
| 3.2 External diagnostics | Costs of external validation of samples |
| 3.3 External non-medical services | Outsourced non-medical services (this is the non-medical counterpart to 3.1 and 3.2) |
| 3.4 Funding of partner organization, not divisible into functions | Note: This category is only used for MSF expenditures classed as 'funding of partner org', where it cannot be categorised as 'external clinical procedures' or 'external diagnostics' or one of the categories under 'sponsor costs'. |
| **4. Purchase of materials** | medical and non-medical |
| 4.1 Medicines and vaccines | Costs of drugs and vaccines used during the clinical trial. This includes both investigational medical product and medicines or vaccines utilized in the standard medical care of patients involved in the trial. |
| 4.2 Medical durables | Clinical trial materials - medical – assets |
| 4.3 Medical consumables (excl. medicines and vaccines) | Clinical trial materials - medical – consumables, which do not fall under 4.1 |
| 4.4 Non-medical durables | Clinical trial materials - nonmedical – assets |
| 4.5 Non-medical consumables | Clinical trial materials - nonmedical – consumables |
| **5. Other** |  |
| 5.1 Share of project cost associated with trial | If the clinical trial is embedded in an ongoing project; a cost estimation for those patients in the project involved in the Trial |
| 5.2 Community engagement | Resources allocated for specific community engagement activities |
| 5.3 Transport and travel | Including transport of staff, medical and non-medical materials, assets, and consumables |
| 5.4 Facility operating costs | Costs associated with building or maintaining clinical trial infrastructure. |
| 5.5 Banking and tax | Financial costs, in particular banking costs and taxes |
| 5.6 Losses, theft, expiries | Losses, theft, and expiries |
| 5.7 Miscellaneous | Miscellaneous |
| **6. Uncategorizable** | Expenditures where details are lacking |

**Notes on cost categorization**

Central staff costs prior to 2017 were allocated to the ‘trial planning’ category.

Central staff costs from 2017 onwards were allocated to the ‘trial management’ category.

**Currency conversion**

Currency conversion was done at the time of preparing internal bookkeeping files, i.e., generally, at most a few months after costs were incurred.

**Methods. Systematic review of available publications on the cost of clinical trials.**

**Introduction**

Clinical trials are a cornerstone of biomedical innovation and evidence-based medicine.^4^ Information gained from clinical trials informs medical decision-making and underpins the regulatory approval of new healthcare products.

Randomised clinical trials are resource-intensive and represent a significant expenditure for research funders. They are widely cited to be the costliest aspect of the pharmaceutical research and development (R&D).^5^ As a result, non-industry clinical trials struggle to obtain sufficient funding and often rely on voluntary activities to achieve their objectives.^6^

Despite their essential role in evidence generation and pharmaceutical research and development, a previous systematic review of publications on costs of randomized clinical trials found that overall cost data remains scarce, with widespread lack of transparency of methodology, and an absence of detailed empirical data on costs of all aspects of randomized clinical trials.^7^ In recent years, there has been renewed attention to the costs of clinical trials and their role in pharmaceutical research and development, therefore an update on the available evidence of clinical trial costs is merited.

**Methods**

We searched for peer-reviewed publications and grey literature that reported or estimated the cost of clinical trials, published between 1974 and 25 May 2023, across the Embase, HMIC Health Management Information Consortium, and Social Policy and Practice databases (Table C).

We excluded publications that only considered one cost component of clinical trials (e.g., source data verification), editorials, commentaries, preprints, and articles not in English, French, German, Spanish, or Portuguese.

All titles and abstracts were screened by two reviewers. For any articles where the reviewers disagreed over inclusion, the full text of the relevant article was jointly and differences in categorization were resolved through discussion. After the screening and discussion, one author reviewed all full-text articles deemed relevant by both reviewers. For each article, reference lists were screened for additional articles of relevance to the systematic review. Overall cost estimates and costs per patient or subject were extracted from each included publication. Additionally, were available, information on clinical trial type, publication year, and analysis on cost drivers were extracted from the articles. We did not adjust figures for inflation.

**Table C. Search strategy.**

The Ovid (Wolters Kluwer) platform was used for the database search, with queries and filters set as outlined below.

Embase <1974 to 2023 May 25>

HMIC Health Management Information Consortium <1979 to March 2023>

Social Policy and Practice <202304>

| Search # | Query | Results |
| --- | --- | --- |
| 1 | (cost* or expen* or budget*).ab. /freq=2 | 438954 |
| 2 | (economic* or cost or costs or costly or costing or price or prices or pricing or pharmacoeconomic* or pharmaco-economic* or expenditure or expenditures or expense or expenses or financial or finance or finances or financed).ti,kw. | 342572 |
| 3 | or/1-2 | 600550 |
| 4 | trial*.ti. | 533735 |
| 5 | (clinical trial* or pharmaceutical trial* or drug trial* or human stud* or clinical stud* or drug stud* or pharmaceutical stud*).ab. | 847781 |
| 6 | or/4-5 | 1255761 |
| 7 | (plan* or budget* or report* or evaluat* or monitor*).ti. | 2257060 |
| 8 | 3 and 6 and 7 | 4554 |
| 9 | remove duplicates from 8 | 4340 |

Exported to Excel spreadsheet and output filtered to remove preprints and articles not in English, French, German, Spanish, or Portuguese.

**Results. Literature identified in the systematic review.**

Twenty-two studies met inclusion criteria (Figure A).

**Figure A. PRISMA flow diagram for literature search.**
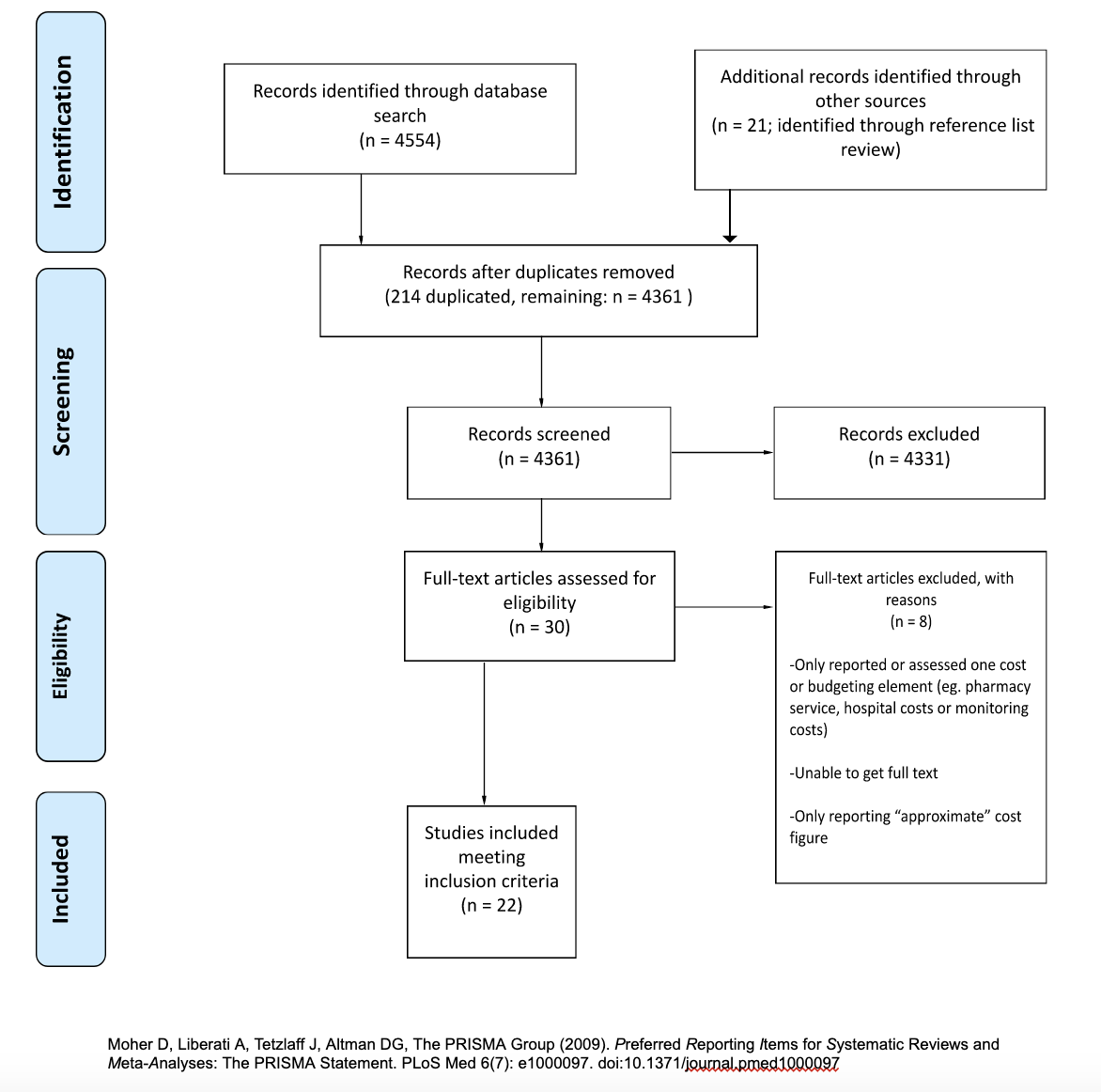


Across the 22 publications (Table D), none reported a detailed empirical analysis of all costs incurred in a specific clinical trial – a fact that was recently highlighted in a similar systematic review.^7^ Some of the identified studies reported averages or ranges for trial costs across numerous trials; further, those studies did not break down (average) costs by expense type, to a granular level. Other identified studies report on modelling exercises to estimate clinical trial costs for various scenarios.

The types of interventions considered in the studies varied. 15 studies concerned pharmaceutical clinical trials, 4 considered vaccine trials, 4 considered trials for diagnostics or prevention tools, and 9 considered clinical management approaches (multiple publications considered multiple types of intervention).

The types of trials covered by the included publications also varied. Although all 22 publications reported on costs for randomised controlled trials, several also included estimates for other types of trials (e.g., pharmacokinetic trials, observational trials). Among the ten publications that assessed the cost of clinical trials in the context of pharmaceutical and vaccine research and development, all 10 providing estimates for phase 3 trials, but only 5 publications containing estimates for the costs of phase 1 or phase 2 trials.

Overall, reported costs for clinical trials ranged from $2,500 (in 2008 USD) to $454 million (in 2011 USD). However, this broad range reflects the wide variation in types of trial, intervention, trial phase, the year the trial(s) took place, and trial duration.

For pharmaceutical development RCTs in phase 1 and 2, the highest and lowest figures out of all the publications came from Sertkaya et al (2016). In this paper, costs ranged $1.4–6.6 million (in 2004-2012 USD) for a phase 1 trial and $7–19.6 million (in 2004-2012 USD) for a phase 2 trial.^8^ Phase 3 pharmaceutical development RCTs costs ranged from $11.5 million (in 2004-2012 USD) to $142 million (in 2005 USD).^8,9^ When restricted to RCTs for phase 2 and phase 3 trials for pharmaceuticals, per trial costs ranged US$5-142 million (USD values as reported in relevant publications, not further inflation adjusted). In order to extract a subset for the purposes of contextualising the costs of TB-PRACTECAL, we identified the range of costs for phase 2 and 3 RCTs for pharmaceuticals, from publications published in the last 20 years (some of the included publications use cost data from the early 1980s which, we would argue, is likely to be outdated for comparison to modern trial costs). For the 5 publications meeting these criteria, we inflation-adjusted costs to 2023 USD. This yielded a range of US$7-221 million per trial, across phase 2 and phase 3 trials for pharmaceuticals.

Two publications considered costs of vaccine research and development trials.^10,11^ Light et al estimated the costs of rotavirus vaccine research and development trials and contain the outliers, both highest and lowest, for research and development trials. The authors estimate phased 1 trial costs as low as $2,500 and phase 3 costs as high as $204,114,000 (in 2008 USD).

Trial durations in the included publications varied greatly, from 20 years for a cancer screening RCT, costing $454 million (in 2011 USD), to just 3 months for a pharmaceutical RCT costing $100,000 (in 2012 USD).^12,13^

For 15 of the included studies, a cost estimate per patient was reported or could be assessed. The highest and lowest clinical trial cost estimates from peer-reviewed publications included remains a previous systematic review which cited cost estimates from $295 to $103,254 per patient (2017 USD).^7^ However, as discussed below, this wide range is driven by methodological issues and outlier estimates.

**Narrative summary**

The large variation in both overall clinical trial cost estimates and per-patient cost estimates in publications identified is notable and a topic of health research and policy interest. The full text review revealed common themes which may help explain the large variation in estimated costs.

The variation in two key cost-driving factors may explain variation in cost estimates: Size of trials – both in terms of number of patients and numbers of centres – and duration of trials. Both these factors are repeatedly cited as being responsible for a significant variation within publications which estimated more than one clinical trial cost.^7,11,12,14–17^ While geographical location of the trial may also be a factor affecting costs, publications commenting on geographical location do not agree on how it affects costs and note the paucity of data in non-high-income countries. ^7,12,14,16^

As demonstrated in the results, the type of intervention and trial design may also have a significant impact on the cost of a trial. For example, vaccine development trials tended to be more expensive than their pharmaceutical counterparts.

Two publications which break down costs by disease area, also find significant variations, only some of which can be explained by size or duration.^8^ This suggests that therapeutic areas may require different resources to conduct clinical trials.

When compared to a similar recent systematic review (Speich et al),^7^ the range of costs identified in this review differed due to a several different methodological choices. First, this review only focussed on publications reporting estimates of overall costs of a clinical trial, while Speich et al collected publications which included only one or several cost elements. Second, this review includes all clinical trials, while Speich et al only includes randomised clinical trials.

Further, the very wide range trial costs identified in Speich et al is created by a few atypical trials as well as the inflation adjustment utilized in the systematic review. The highest reported cost ($611 million per trial) is based on an estimated figure of $110 million reported in a 1989 paper (which is then inflation-adjusted by Speich et al).^18^ This paper utilized a mathematical model based on a 1985 paper, which in turn utilized intransparent empirical data from 1975-1985.^19^ Both papers are also included in this systematic review, but due to the decision not to adjust for inflation, these estimates do not represent outliers. Also, the lowest cost estimates for per patient costs are driven by outliers. The lowest estimate for per-patient cost in this systematic review – as well as the previous systematic review if commentaries are excluded – comes from Anguera et al, which utilized a very low-cost remote technology-based intervention in a psychiatric clinical trial.^20^

**Table D. Table of characteristics of publications included in the systematic review**

| **Reference** | **Year** | **Type of trials** | **Cost per patient (inflation-adjusted figures, where applicable, in italics)*** | **Total cost per trial (inflation-adjusted figures, where applicable, in italics)*** |
| --- | --- | --- | --- | --- |
| Park et al^21^ | 2022 | Cost estimates for platform, multi-group, and adaptive trials based on a single pharmaceutical RCT. Trial phase not clear or unspecified. | ~$225,000 | ~$100 million |
| Moore et al^15^ | 2020 | Phase 3 pharmaceutical clinical trials | $41,413 USD [*$48,756 USD*] | Median estimate $19 million (2020 USD) [*$22.4 million USD*]. |
| Moore et al^14^ | 2018 | Pharmaceutical RCTs phase 3 | “Estimated costs increased from a mean of $5.9 million [*$7.2 million*][for] 8 trials enrolling 100 or fewer patients to a mean of $77.2 million [*$94 million*][for] 21 trials enrolling more than 1000 patients”. | Median estimated cost of $19.0 million current (2018 USD) [*$23.1 million USD*]. |
| Martin et al^16^ | 2017 | Phase 1, 2 and 3 pharmaceutical clinical trials | N/A | Phase 1: $3.4 million (2017 USD)  Phase 2: $8.6 million (2017 USD)[*$10.7 million*]  Phase 3: $21.4 million (2017 USD)[*$26.6 million*] |
| Pham et al^22^ | 2017 | Perinatal RCTs | AUD $590- 16,689 | AUD $14,167 to AUD $1,785,714 (2016 AUD) |
| Speich, Niederhäusern, Blum et al^12^ | 2017 | 2 pharmaceutical RCTs. Trial phase not clear or unspecified. | $208-$2,868 in 2012 USD. | $0.1–2.3 million (current 2012 USD). |
| Speich, Niederhäusern, Shur et al^7^ | 2017 | Systematic review of articles assessing overall RCT costs.  43.8% were in the medical field, 6.3% in surgery, and 31.3% other (unclear in the remaining 18.8%).  Mixture of RCTs with trial phases 2-4 and RCTs with unspecified trial phases. | $295-103,254  82 per patient in 2017 USD | $0.2-611.5 million per RCT, adjusted to 2017 USD. |
| Sertkaya et al^8^ | 2016 | Phase 1, 2, and 3 pharmaceutical clinical trials | N/A | Phase 1 trial: $1.4–6.6 million (current 2004-2012 USD)  Phase 2:  $7–19.6 million (current 2004-2012 USD)[*$9.9-27.7 million*]  Phase 3: $11.5-52.9 million in (current 2004-2012 USD)[*$16.3–74.9 million*]  (costs are also reported by disease category but not reproduced here) |
| Larson et al^23^ | 2016 | 3 pharmaceutical & medical management RCTs. Trial phases not clear or unspecified. | $17,313- $20,441 USD | $71,850,000–111,855,000 (current 2004 USD) |
| Anguera et al^20^ | 2016 | Remote app based psychiatric RCT. Trial phase not clear or unspecified. | $286 | $314,264 (current USD 2014-2015) |
| Redmond et al^24^ | 2013 | Pediatric pharmaceutical RCT. Trial phase not clear or unspecified. | N/A | £697,135 (current 2005-2007 GBP) |
| Chit et al^10^ | 2013 | Vaccine phase 3 trial | $11,515-20,282 | Phase 1: $1,919,944 Phase 2: $10,485,647 Phase 3: $51,368,604 (2022 CAD). |
| Zhu et al^13^ | 2013 | Cancer screening RCT of 20-year duration. Trial phase not clear or unspecified. | N/A | $454 million (inflation-adjusted to 2011 USD) |
| Raftery et al^17^ | 2011 | 125 RCTs - 19.3% Diagnostic and screening - 22.9% pharmaceutical - 56% Therapeutic procedures  5.7% where pharmaceutical development 17.9% pharmaceutical evaluation  Trial phases not clear or unspecified. | N/A | 0.25 to 2.5 million GBP (current GBP 1995–2005). |
| Light et al^11^ | 2009 | Vaccine development RCT | Phase 1: $100-$400 Phase 2: $300-400 Phase 3: $2,000-3,000 | Phase 1: $2,500-$130,000 Phase 2: $55,200-$985,600 Phase 3: $126,450,000 – $204,114,000  (2008 USD) |
| Baker-Smith et al^25^ | 2008 | RCT to obtain pediatric exclusivity. | $7,605-37,291 | $4.3 million (range $2.1–12.9 million) for safety and efficacy studies; $862,000 (range: $556,000–1.8 million) for pharmacokinetic studies. (current 1997-2004 USD) |
| Li et al^26^ | 2007 | RCT to obtain pediatric exclusivity. Phase 4 trial. | $14,044-75,686 | Median efficacy study cost was $6,464,921 ($1,770,566–$12,948,325) (2005 USD) |
| Johnston et al^27^ | 2006 | Neurological clinical trials. Mixture of therapies, prevention, practices. Trial phases not clear or unspecified. | N/A | $722,105–64,033,234 (current USD 1997–2007) |
| Eisenstein et al ^9^ | 2005 | Modelled costs for two hypothetical phase 3 cardiovascular pharmaceutical RCTs, using experts’ assumptions | $21,366-$33,066  [*$36,781-$56,922*] | $142 million (range $102 to $207 million) (current 2001 USD) for a double-blind chronic heart failure trial [*$244 million; range $175-356 million*];  $83 million (range $57 to $158 million) (current 2001 USD)for an open-label acute coronary syndrome trial [*$143 million; range $98-272 million*]. |
| Drummond et al^28^ | 1992 | RCT of photocoagulation of proliferative diabetic retinopathy | $6,062 | $10.5 million (1982 USD) |
| Detsky et al^18^ | 1989 | Pharmaceutical, prevention, and medical and surgical management trials | N/A | $78,000-$150,000,000 (date of USD value not specified) |
| Detsky et al^19^ | 1985 | Two phase 4 pharmaceutical RCTs | $3,125-4,250 | $5-$17 million (1975-1985 USD) |

*Costs for pharmaceutical phase 2 and phase 3 studies published within the last 20 years are reported as in the source publication with figures which are currency converted and inflation adjusted to March 2023 using <https://www.usinflationcalculator.com>, using publication date as the base year, unless cost periods are otherwise specified in the publication. Costs for studies older than 20 years are reported as in the source publication, without currency conversion or inflation adjustment (unless already adjusted in the source).

**Table E. Per-patient costs.**

| **Site** | **Mean cost per patient (site-specific costs only)** | **Mean global costs per patient (site-specific plus central costs)** |
| --- | --- | --- |
| Minsk | €19,998 | €44,132 |
| Karakalpakstan | €45,942 | €70,076 |
| Tashkent | €39,510 | €63,644 |
| THINK | €41,081 | €65,215 |
| Wits | €21,145 | €45,279 |
| Overall | **€37,326** | **€61,460** |

**Table F. Costs by category, per site, per patient (EUR).**

| **Category heading** | **Category** | **Minsk** | **Nukus/Karalpakstan** | **Tashkent** | **THINK** | **Wits** | **Average** |
| --- | --- | --- | --- | --- | --- | --- | --- |
| External services supporting work at clinical trial sites | External clinical procedures | €2,622 | €0 | €101 | €830 | €0 | €725 |
|  | External diagnostics | €5 | €1 | €84 | €7,967 | €7,845 | €2,959 |
|  | External non-medical services | €113 | €0 | €516 | €75 | €0 | €94 |
|  | Funding of partner organization, not divisible into functions | €0 | €1,550 | €0 | €0 | €0 | €542 |
| Other | Banking and tax | €0 | €0 | €0 | €45 | €0 | €13 |
|  | Community engagement | €49 | €0 | €70 | €7 | €129 | €28 |
|  | Facility operating costs | €185 | €650 | €251 | €1,133 | €676 | €671 |
|  | Losses, theft, expiries | €44 | €39 | €25 | €0 | €0 | €24 |
|  | Misc | €0 | €141 | €0 | €3,757 | €807 | €1,220 |
|  | Transport and travel | €1,235 | €3,625 | €4,016 | €495 | €120 | €2,045 |
| Purchase of materials (medical and non-medical) | Medical consumables (excl. medicines and vaccines) | €634 | €7,913 | €3,419 | €114 | €152 | €3,266 |
|  | Medical durables | €610 | €1,056 | €1,375 | €124 | €0 | €652 |
|  | Medicines | €4,725 | €3,931 | €4,121 | €4,261 | €4,261 | €4,215 |
|  | Non-medical consumables | €379 | €1,343 | €234 | €423 | €0 | €686 |
|  | Non-medical durables | €147 | €85 | €947 | €1,188 | €0 | €501 |
| Trial site staff costs | Trial site staff (specifically contracted) | €9,226 | €25,588 | €24,317 | €20,664 | €6,938 | €19,654 |
| Uncategorizable | Uncategorizable | €0 | €0 | €0 | €0 | €0 | €0 |

Highlights in blue indicate above-average costs.

**Table G. Costs by category, per patient, as percent above/below the average.**

|  |  | Minsk | Karakalpakstan | Tashkent | THINK | Wits |
| --- | --- | --- | --- | --- | --- | --- |
|  |  |  |  |  |  |  |
| External services supporting work at clinical trial sites | External clinical procedures | +262% |  | -86% | +14% |  |
|  | External diagnostics | -100% | -100% | -97% | +169% | +165% |
|  | External non-medical services | +20% |  | +450% | -20% |  |
|  | Funding of partner organization, not divisible into functions |  | +186% |  |  |  |
| Other | Banking and tax | -100% |  |  | +239% |  |
|  | Community engagement | +78% |  | +154% | -75% | +365% |
|  | Facility operating costs | -72% | -3% | -63% | +69% | +1% |
|  | Losses, theft, expiries | +83% | +62% | +6% |  |  |
|  | Misc |  | -88% |  | +208% | -34% |
|  | Transport and travel | -40% | +77% | +96% | -76% | -94% |
| Purchase of materials (medical and non-medical) | Medical consumables (excl. medicines and vaccines) | -81% | +142% | +5% | -97% | -95% |
|  | Medical durables | -7% | +62% | +111% | -81% |  |
|  | Medicines | +12% | -7% | -2% | +1% | +1% |
|  | Non-medical consumables | -45% | +96% | -66% | -38% |  |
|  | Non-medical durables | -71% | -83% | +89% | +137% |  |
| Trial site staff costs | Trial site staff (specifically contracted) | -53% | +30% | +24% | +5% | -65% |

**Table H. Top 10 trial medicines by total cost.**

| **Medicine** | **Cost (EUR)** | **Cost* (%)** |
| --- | --- | --- |
| bedaquiline | 667,318 | 45.6% |
| linezolid | 234,851 | 16.0% |
| imipenem/cilastatin | 150,227 | 10.3% |
| delamanid | 125,379 | 8.6% |
| clofazimine | 59,637 | 4.1% |
| capreomycin | 49,483 | 3.4% |
| cycloserine | 31,712 | 2.2% |
| moxifloxacin | 16,058 | 1.1% |
| saline | 16,040 | 1.1% |
| pretomanid | 14,564 | 1.0% |

*percentage of total medicines expenditure in the covered sites.

Data covers Uzbekistan and Belarus, representing 63% of enrolled patients. Data broken down by individual medicines was not available for South Africa.

**Bibliography**

1. Médecins Sans Frontières. MSF Clinical Trial Transparency Policy. Published November 11, 2022. https://msfaccess.org/msf-clinical-trial-transparency-policy

2. DiMasi JA, Grabowski HG, Hansen RW. Innovation in the pharmaceutical industry: New estimates of R&D costs. *J Health Econ*. 2016;47:20-33. doi:10.1016/j.jhealeco.2016.01.012

3. Ariella Barel, Laurel Boman, Christopher Morten. Clinical Trial Cost Transparency at the National Institutes of Health: Law and Policy Recommendations. NYU School of Law. Published August 2020. https://www.law.nyu.edu/sites/default/files/Clinical_Trial_Cost_Transparency_at_the_NIH-Law_and_Policy_Recommendations.pdf

4. Siepmann T, Spieth PM, Kubasch AS, Penzlin AI, Illigens BMW, Barlinn K. Randomized controlled trials &ndash; a matter of design. *Neuropsychiatr Treat Publ Online June*. 2016(1341). doi:10.2147/NDT.S101938

5. Morgan S, Grootendorst P, Lexchin J, Cunningham C, Greyson D. The cost of drug development: A systematic review. *Health Policy*. 2011;100(1):4-17. doi:10.1016/j.healthpol.2010.12.002

6. Djurisic S, Rath A, Gaber S. Barriers to the conduct of randomised clinical trials within all disease areas. *Trials*. 2017;18(1). doi:10.1186/s13063-017-2099-9

7. Speich B, Niederhäusern B, Schur N. Systematic review on costs and resource use of randomized clinical trials shows a lack of transparent and comprehensive data. *J Clin Epidemiol*. 2018;96:1-11. doi:10.1016/j.jclinepi.2017.12.018

8. Sertkaya A, Wong HH, Jessup A, Beleche T. Key cost drivers of pharmaceutical clinical trials in the United States. *Clin Trials*. 2016;13(2):117-126. doi:10.1177/1740774515625964

9. Eisenstein EL, Lemons PW, Tardiff BE, Schulman KA, Jolly MK, Califf RM. Reducing the costs of phase III cardiovascular clinical trials. *Am Heart J*. 2005;149(3):482-488. doi:10.1016/j.ahj.2004.04.049

10. Chit A, Parker J, Halperin SA, Papadimitropoulos M, Krahn M, Grootendorst P. Toward more specific and transparent research and development costs: The case of seasonal influenza vaccines. *Vaccine*. 2014;32(26):3336-3340. doi:10.1016/j.vaccine.2013.06.055

11. Light DW, Andrus JK, Warburton RN. Estimated research and development costs of rotavirus vaccines. *Vaccine*. 2009;27(47):6627-6633. doi:10.1016/j.vaccine.2009.07.077

12. Speich B, Niederhäusern B, Blum CA. Retrospective assessment of resource use and costs in two investigator-initiated randomized trials exemplified a comprehensive cost item list. *J Clin Epidemiol*. 2018;96:73-83. doi:10.1016/j.jclinepi.2017.12.022

13. Zhu CS, Pinsky PF, Kramer BS. The Prostate, Lung, Colorectal, and Ovarian Cancer Screening Trial and Its Associated Research Resource. *JNCI J Natl Cancer Inst*. 2013;105(22):1684-1693. doi:10.1093/jnci/djt281

14. Moore TJ, Zhang H, Anderson G, Alexander GC. Estimated Costs of Pivotal Trials for Novel Therapeutic Agents Approved by the US Food and Drug Administration, 2015-2016. *JAMA Intern Med*. 2018;178(11). doi:10.1001/jamainternmed.2018.3931

15. Moore TJ, Heyward J, Anderson G, Alexander GC. Variation in the estimated costs of pivotal clinical benefit trials supporting the US approval of new therapeutic agents, 2015–2017: a cross-sectional study. *BMJ Open*. 2020;10(6). doi:10.1136/bmjopen-2020-038863

16. Martin L, Hutchens M, Hawkins C, Radnov A. How much do clinical trials cost? *Nat Rev Drug Discov*. 2017;16(6):381-382. doi:10.1038/nrd.2017.70

17. Raftery J, Young A, Stanton L. Clinical trial metadata: defining and extracting metadata on the design, conduct, results and costs of 125 randomised clinical trials funded by the National Institute for Health Research Health Technology Assessment programme. *Health Technol Assess*. 2015;19(11):1-138. doi:10.3310/hta19110

18. Detsky AS. Are Clinical Trials a Cost-effective Investment? *JAMA*. 1989;262(13):1795-1800. doi:10.1001/jama.1989.03430130071037

19. Detsky AS. Using economic analysis to determine the resource consequences of choices made in planning clinical trials. *J Chronic Dis*. 1985;38(9):753-765. doi:10.1016/0021-9681(85)90118-3

20. Anguera JA, Jordan JT, Castaneda D, Gazzaley A, Areán PA. Conducting a fully mobile and randomised clinical trial for depression: access, engagement and expense. *BMJ Innov*. 2016;2(1):14-21. doi:10.1136/bmjinnov-2015-000098

21. Park JJH, Sharif B, Harari O. Economic Evaluation of Cost and Time Required for a Platform Trial vs Conventional Trials. *JAMA Netw Open*. 2022;5(7). doi:10.1001/jamanetworkopen.2022.21140

22. Pham CT, Karnon JD, Middleton PF. Randomised clinical trials in perinatal health care: a cost‐effective investment. *Med J Aust*. 2017;207(7):289-293. doi:10.5694/mja16.01178

23. Larson GS, Carey C, Grarup J. Lessons learned: Infrastructure development and financial management for large, publicly funded, international trials. *Clin Trials*. 2016;13(2):127-136. doi:10.1177/1740774515625974

24. Redmond NM, Hollinghurst S, Costelloe C. An evaluation of the impact and costs of three strategies used to recruit acutely unwell young children to a randomised controlled trial in primary care. *Clin Trials*. 2013;10(4):593-603. doi:10.1177/1740774513494503

25. Baker-Smith CM, Benjamin DK, Grabowski HG. The economic returns of pediatric clinical trials of antihypertensive drugs. *Am Heart J*. 2008;156(4):682-688. doi:10.1016/j.ahj.2008.05.001

26. Li JS, Eisenstein EL, Grabowski HG. Economic Return of Clinical Trials Performed Under the Pediatric Exclusivity Program. *JAMA*. 2007;297(5). doi:10.1001/jama.297.5.480

27. Johnston SC, Rootenberg JD, Katrak S, Smith WS, Elkins JS. Effect of a US National Institutes of Health programme of clinical trials on public health and costs. 2006;367.

28. Drummond MF, Davies LM, Ferris FL. Assessing the costs and benefits of medical research: The diabetic retinopathy study. *Soc Sci Med*. 1992;34(9):973-981. doi:10.1016/0277-9536(92)90128-D
