# Supplementary material for: Cost analysis of the TB-PRACTECAL clinical trial on novel tuberculosis treatment regimens
Source: PLOS Glob Public Health. 2025 Apr 23;5(4):e0003759. doi: 10.1371/journal.pgph.0003759 (PMC12017904; doi:10.1371/journal.pgph.0003759)
Supplement: Attachment — Submitted filename: PRACTECAL cost paper PLOS GPH response to review 2024-09-04.docx [file pgph.0003759.s002.docx]

Dear Editor and Reviewers,

Thank you for your review of this manuscript. We have addressed all comments, as outlined below.

With kind regards,

Dr Dzintars Gotham on behalf of all authors

***

EDITOR COMMENTS:

The manuscript is well written and could transform the way trials are reported. Transparency about trial costs could lower drug prices and foster public-private partnerships in funding these studies.
However, the introduction lacks information and literature references, and the methodology is weakly presented. It is recommended to thoroughly reinforce the introduction and methodology before submission.

AUTHOR RESPONSE:

Thank you for the recommendations.

We have addressed these recommendations with detailed responses within Reviewer #2’s comments below. We have added further details to the text in Methods, as well as ensuring that text on the search strategy for the literature review is no longer in the appendix but presented in the main text. The extent of the edits recognise that our study is not a cost-effectiveness or an economic evaluation but a descriptive cost analysis study.

Please see also more detailed responses to these points below, in responses to Reviewer #2.

REVIEWER COMMENT:

Reviewer #1: The study by Gotham et al is an analysis costs of the TB-PRACTECAL clinical trial on novel tuberculosis treatment regimens. This is an important study that sheds light on the costs and the major drivers of costs that are incurred during phase 2b-3 clinical trial to inform pricing of drugs. Overall, the manuscript is well written and could be a game changer in the way trials are reported. Openness about costs of trials may lower costs of drugs and foster Public Private participation in funding trials. The authors show that it is possible to keep trial cost at low levels despite long duration of trial, high cost of drugs used in the trial e.g. bedaquiline and extensive laboratory/facility investments that may be required in some settings. Congratulations to the authors.

AUTHOR RESPONSE:

We thank Reviewer #1 for their kind words and thoughtful comments.

REVIEWER COMMENT:

Setting of the trial obviously influences the costs. In developed countries, the major cost drivers are HR costs, but in developing countries costs are pushed up due to setting up specialised facilities. Based on these results, what do authors suggest is the best way possible to lower the cost drivers? Perhaps the authors may make it clear that setting up trials in middle income countries even out health disparities and economies of scale can be realised in the long run if more trials are done. Openness of costs incurred during trials may foster collaborations by other players e.g. governments, NGOs, academia and pharmaceutical industry to lower costs further.

AUTHOR RESPONSE:

Thank you for these insightful points.

We agree that pre-existing health infrastructure influences the site costs of conducting clinical trials. We however are not able to authoritatively comment on approaches to lower clinical trial cost drivers based on the data from our study. Increased sharing of granular clinical trial cost data, as the reviewer alludes, could perhaps help to identify where costs can be reduced.

We certainly agree with the points regarding the need for greater clinical trial infrastructure in LMICs and the likely ‘economies of scale’ that can be reached once infrastructure is established. We have added text to capture this point, in the Discussion, in lines 294-299.

REVIEWER COMMENT:

Attrition from the trial can have an impact on the costs. People who drop from the trial at the beginning (lower costs) than those who drop out around the end of the trial. Based on this, what was the % of participants who dropped out from the trial? S1 TABLE may be edited to reflect the number of participants that were enrolled/finished the trial.

AUTHOR RESPONSE:

Indeed, it makes sense to mention the attrition rate. Thank you for suggesting this.

We have used the enrolment number (552 patients) for our analysis of average costs per patient. The intention-to-treat population included 550 patients. The per-protocol population was 410 patients i.e. 410 patients completed the entire trial protocol (among other things, 68 patients discontinued the treatment early, and 13 patients withdrew consent prior to completing trial). The overall attrition rate was thus 26%.

However, as discontinued patients could still be followed up to week 108 if they didn’t withdraw consent and those follow-up costs are reflected in the trial’s total expenditures, using the numbers recruited felt to be the most relevant reference point.

We have added clarification to explicitly recognise the attrition on lines 229-231.

REVIEWER COMMENT:

Discussion:
Line 334: Based on the analysis of TB-PRACTECAL costs, we developed a standardized list of reportable cost categories (S2 Table). Is there scope to include costs that are associated with treatment/management of adverse events in S2 Table?

AUTHOR RESPONSE:

Thank you for this interesting point. We recognise that the cost of managing adverse effects has been the subject of several analyses of clinical trial costs and that it is an important aspect in economic evaluation and cost-effectiveness studies.

As we outline in the manuscript, the cost category list that we developed is a balance between practical ease of use, granularity, and being analytically useful. We expect that the great majority or perhaps all research organizations will not be separating ‘regular’ clinical follow-up from ‘adverse event’ management in their bookkeeping – as such, significant additional estimation would be required to differentiate these two elements.

Including the clinical management of adverse effects as a separate category would require

a. breaking down the clinical management in general into different components (e.g. deciding what proportion of staff time (categories 2.1 and 2.2) was dedicated to regular trial activities and what proportion was dedicated to managing adverse effects.
b. Similarly, allocating the costs of medical durables and consumables into proportions.
c. Similarly, allocating the costs of medicines (granted, in some trials this may be straightforward, e.g. antiemetics in oncology trials).

For the present study, especially with the second objective of looking at feasibility of routine clinical trial cost reporting, we considered the current granularity of reporting sufficient.

We have added this into the Limitations section (lines 402-404). Additionally, we will raise this in ongoing work we are undertaking with multiple partners to develop generalized clinical trial cost reporting guidelines.

REVIEWER COMMENT:

The text in Fig 1 is not very clear. May the authors upload files that have a high resolution.
May the authors use the referencing style where in text in in square brackets [].

AUTHOR RESPONSE:

Figure 1 is uploaded in high resolution. We are aware that the Figure does not render very clearly in the manuscript PDF generated for peer reviewers by PLOS. A full-definition figure can be downloaded from the link in the upper-right corner of the page where the Figure is presented, in the peer review PDF. We will ensure that a high-resolution Figure is included in the published version, if accepted for publication.

Thank you for the note regarding presenting citations in square brackets. We have fixed this in the manuscript.

REVIEWER COMMENT:

Reviewer #2: Selected topic is interesting but lacking much information and literature in introductory chapter and very much weak presentation in methodology chapter extremely disappointed us. So, many relevant literature should be added to the introductory chapter with subtle references. Methodology is very vital chapter and issue of any empirical or secondary research. This manuscript has focused on discussion chapter rather than methodological areas/rooms ( Such as, study design/approach, study settings, population and sample, data processing/data collection and analysis and so on). Both "Introduction and methods" have been presented in very laconic way which undermined this manuscript.

AUTHOR RESPONSE:

We thank Reviewer #2 for their comments.

We certainly agree that the manuscript should clearly and thoroughly present the relevant background literature and the methods used in the analysis. We are eager to improve the manuscript where possible to meet these aims.

Reviewer 2# “the introduction is lacking information and that references should be added”:

The introduction cites 18 references. We believe that we have cited all relevant literature, these were identified in our literature review and in workshops we have held with experts. The paucity of literature in this field is another reason we feel our paper has potential to add to this gap.

Reviewer 2# recommended the following elements in the Methods: study design/approach, study settings, population and sample, data processing/data collection and analysis.

For the ‘study design/approach’:

We are not aware of previously defined and published reporting standards for a clinical trial cost analysis, we therefore included study design elements that were arrived at after consulting a limited number of experts. We recognise the need for such standards to be defined and hence consider this as a first step. We have added a sentence summarizing the ‘study type’ as the first sentence of Methods (line 107).

The study setting is described under ‘Expenditure data’ (as well as the Introduction), where the manuscript explains the time period, trial sites, enrolment number, etc. We have edited Methods to reiterate certain details presented in the Introduction (lines 109-115).

For ‘data processing/data collection and analysis’:

This is outlined under the subheadings ‘Expenditure data’ and ‘Cost categorization and analysis’. We have added further details under these headings (lines 117-168). We have also moved text on the details of the systematic literature review including the search strategy from the Appendix to the Methods in the main text (lines 178-190).
