# Supplementary material for: Cost analysis of the TB-PRACTECAL clinical trial on novel tuberculosis treatment regimens
Source: PLOS Glob Public Health. 2025 Apr 23;5(4):e0003759. doi: 10.1371/journal.pgph.0003759 (PMC12017904; doi:10.1371/journal.pgph.0003759)
Supplement: Attachment — Submitted filename: Review Comments.docx [file pgph.0003759.s003.docx]

**Review Comments**

**General Comment**

It is commendable that the authors are trying to encourage transparency regarding the disclosure of the costs of drug development that are also the major drivers of the price of drugs. The results are also analyzed and described in sufficient detail.

However, there are still issues that require revision for considering the manuscript publishable.

The claim that the study has standardized cost analysis methods is not justified. There are already established parameters for health services cost analysis, and these can easily be customized for costing clinical trials.

In addition, it would not be fair to consider there has been proper categorization of the cost items – with 2% and 9% of the items labeled as “uncategorizable” and “other” respectively.

**Specific Comments**

- **Methods**
  - The study should explicitly state the population being studied and the type of health facility where the research was conducted. This will help contextualize the findings.
  - Study should show whether the costing followed step-down allocation or ingredient approach. Along with that, how data were obtained should be explained.
  - Systematic review sub-section (lines 170-189) – This sub-section should be removed as it is too much detail. It only suffices to mention that the authors have done a systematic review on the subject.
  - The methods section also lacks proper description of the statistical analysis employed. This should include how the mean cost was calculated, which cost drivers were included, and details on sensitivity analysis and cost discounting.
- **Results**
  - Results derived from the systematic review should either be removed or briefly integrated under the methods section of the manuscript. If they have to be part of the study, the standard guidelines of reporting systematic reviews should be followed.
  - Tables should be self-explanatory. In addition, there should be some text between consecutive tables.
  - Table 3 - How does ‘miscellaneous” differ from “uncategorizable”?
  - “The per-patient averages presented here are based on the number enrolled at the start of the trial” (lines 229-230) – Cannot these be adjusted per the effective “actual participation-equivalents” for the patients? Otherwise, the cost per-patient would be somewhat deflated.
  - “No other medicines were donated for use in the trial” (lines 239-240) – I am not sure about the implication of this in the costing process – All costs have to be included – whether the drugs are obtained through donation or purchase.
- **Discussion**
- The discussion section seems a bit too long in comparison to the results section, and there few of the paragraphs (especially the items between lines 308 and 347) do not emanate from (or are not relevant to) any of the findings of the costing exercise. Furthermore, even though MSF is known an already established NGO at international level, I do not think an article such as this should be used as a forum for promoting its activities. Rather, the authors should discuss potential biases arising from MSF acting as manager, funder, and implementer.
  - “Overall, we conclude that MSF was able to keep costs at an average-to-low level’ (lines 308-309) – I am not sure if such a conclusion is justified when the central monitoring cost of the trials is more than a quarter of all the expenditures.
- **Other/minor Comments**
  - There are some editorial issues that need correction, and authors should also note that “data” are plural.
